# Dual-functional Ni and Co oxide-doped carbon nanocomposite: an effective catalyst for electrochemical water splitting and CO_2_ utilization

**DOI:** 10.1039/d5ra04999g

**Published:** 2025-09-10

**Authors:** Misbah Zia, Zahoor Ahmad, Khurram S. Munawar, Zafar A. K. Khattak, Hamid Raza, Hira Idrees, Hussain A. Younus, Muhammad Ashraf Shaheen, Nazir Ahmad

**Affiliations:** a Institute of Chemistry, University of Sargodha Sargodha-40100 Pakistan; b Department of Chemistry, Government College University Lahore Lahore-54000 Pakistan dr.nazirahmad@gcu.edu.pk; c Department of Chemistry, Faculty of Science, Fayoum University Fayoum 63514 Egypt hay00@fayoum.edu.eg; d Department of Chemistry, Faculty of Science, University of Engineering and Technology Lahore Pakistan; e Department of Chemistry, University of Mianwali Mianwali 42200 Pakistan; f Department of Chemistry, University of Buner Swari Buner 19281 Pakistan; g Department of Chemistry, University of Management and Technology Lahore 54770 Pakistan; h Nanotechnology Research Center, Sultan Qaboos University P. O. Box 17, 123, Al-Khoud Oman; i Department of Allied Health Sciences, Superior University Sargodha-Campus 40100 Pakistan

## Abstract

The catalytic activation of small molecules is an excellent approach for scientific and technological developments. The production of green hydrogen and fine chemicals through water splitting and carbon dioxide fixation, respectively, is highly effective and eco-friendly; it can meet the requirements for energy economy and sustainability. Transition metal-derived nanomaterials are considered very efficient catalysts. Herein, we developed a metal oxides@carbon (NCC) nanocomposite derived from a bimetallic (Ni/Co) MOF as a multifunctional catalyst. The NCC catalyst was successfully investigated for CO_2_ fixation, oxygen evolution, and hydrogen evolution reactions. The NCC nanocomposite catalyst shows a noticeable CO_2_ cycloaddition to epoxide efficiency of 74–99.9% at 50–100 °C under 1.97 atm in 24 h. NCC exhibits low overpotentials (*η*_10_) for an alkaline (1 M KOH) medium OER and HER, *i.e.*, 310 and 200 mV with Tafel slopes of 76 and 117 mV dec^−1^, respectively. Similarly, for an acidic (0.1 M H_2_SO_4_) medium HER, *η*_10_ = 118 mV and Tafel slope = 47 mV dec^−1^. High electron conductivity with very low charge transfer resistance is observed (*R*_ct_/Ω = 0.55 for the OER_alkaline_, 8.13 for the HER_alkaline_, and 0.824 for the HER_acidic_). Bulk electrolysis revealed stable performance for 10–15 h at *η*_10_ in each case without any major changes in the structural morphology of NNC. These results show the synergy of the active sites for achieving superior catalytic properties, presenting NCC as a suitable candidate for sustainable energy applications.

## Abstract 2

An MOF-derived NiO_*x*_/Co_*y*_O_*z*_-doped carbon (NCC) nanocomposite is synthesized and effectively used as a multifunctional and robust catalyst. Precious cyclic carbonates and green H_2_ are produced *via* small-molecule activation methodologies.

## Graphical abstract

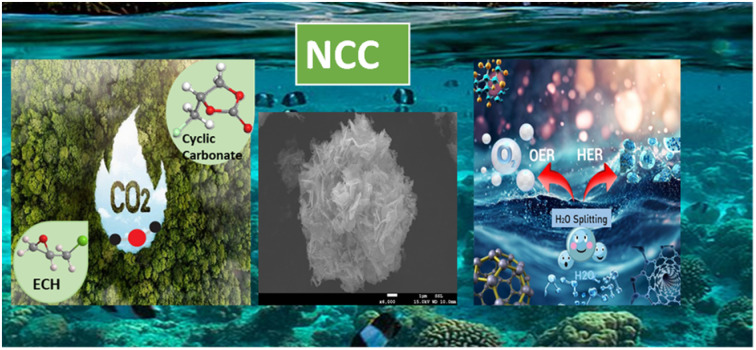

## 1. Introduction

The demand for global energy is increasing day by day, and fossil fuel burning is the major source of energy. The combustion of fossil fuels is a major contributor to meet our energy requirements, but it generates greenhouse gases that drastically negatively affect human health and global warming.^1^ Many alternative energy sources are available, such as hydrothermal, wind, solar, and tidal energies, but their operational condition limits their use. The generation of green energy and fine chemicals through small-molecule activation is an outstanding scientific and technological development. Small molecules such as water and carbon dioxide have driven the interest of researchers to generate green hydrogen and useful chemical products, *i.e.*, cyclic carbonates, methane, formic acid, methanol, *etc.*^2^ Cyclic carbonates have extensive applications as solvents and electrolytes as well as in cosmetics, polymers, and medicines.^3,4^ The cycloaddition of CO_2_ to epoxides is 100% environmentally friendly as compared to other conventional methods that cause the production of toxic and corrosive gases.^5^ In line with CO_2_ fixation, electrochemical water splitting is an effective approach for energy conversion/production. Water splitting activity is based on two mechanisms, *i.e.*, the OER and HER in two half-cells that cause green hydrogen production.^6,7^ Catalysts based on noble metals as electrochemical catalysts are efficient for water splitting at low overpotential. However, these catalysts are costly and economically unsuitable.^8^ Hence, there is a need for cost-friendly and efficient catalysts to avoid sluggish OER kinetics with a relatively low energy barrier. For this purpose, catalysts based on earth-abundant transition metals (Mn, Fe, Co, Ni, Cu, *etc.*) are emerging as a viable option that is extensively explored for the OER and HER.^9–11^ The porous heterogeneous catalysts for CO_2_ fixation and water splitting include ionic liquids, porous organic polymers, zeolites, covalent-organic frameworks, metal–organic frameworks, and carbon composites.^12,13^ Among these, MOFs are good candidates for CO_2_ cycloaddition due to their high porosity and CO_2_ adsorption.^14,15^ MOFs are directly synthesized by simply reacting the metal source with ligands under certain conditions.^16^ Unfortunately, the poor thermal, chemical, and water stability properties of MOFs restrict their applications.^17^ Therefore, the development of easily separated and recycled catalysts with stable catalytic performance is highly desired. For this purpose, the direct carbonization of MOFs is a useful method to obtain highly porous carbon-based nanocomposites. MOF-derived metal oxides embedded carbon composites generally exhibit large surface area, nanoscale pore size, and active morphologies.^18,19^ MOF-derived metal oxides take advantage of the synergistic effects from the two types of functional components; thus, they not only retain excellent structural characteristics of the framework materials, but also can possess excellent conductive and catalytic properties.^20,21^ This exclusive configuration of the metal oxides@carbon hybrid nanocomposite provides metallic or metal oxide nanoparticles for catalysis and prevents the aggregation of metal nanoparticles at high temperatures.^22^ Herein, a bimetallic nanocomposite (NCC) is prepared from cobalt and nickel derived from a bimetallic MOF (CoNiBDC). The catalytic chemical fixation of CO_2_ is investigated by cycloaddition of CO_2_ to epichlorohydrin, along with electrocatalytic OER and HER in two different media (alkaline and acidic).

## 2. Experimental

### 2.1 Materials

The chemicals Ni(NO_3_)_2_·6H_2_O (Sigma-Aldrich), *N*,*N*′-dimethyl formamide (Sigma-Aldrich), 1,4-benzenedicarboxylic acid (Sigma-Aldrich), sodium hydroxide, Co(NO_3_)_2_·6H_2_O (UNICHEM), methanol (LABSOLV), and distilled water of analytical grade were purchased and used without purification.

### 2.2 Synthesis of materials

To prepare the NiCoBDC MOF, metal salts (*i.e.*, Ni (NO_3_)_2_·6H_2_O (76 mg, 0.26 mmol) and Co(NO_3_)_2_·6H_2_O (76 mg, 0.26 mmol)) and H_2_BDC (83 mg, 0.5 mmol) were dissolved in 10 mL DMF with continuous stirring for 15 minutes to get the homogenous mixture. NaOH (0.4 M, 1 mL) was added to the prepared mixture, and the solution mixture was transferred to a Teflon-lined autoclave (20 mL). The autoclave was heated in an oven at 120 °C for 15 h. After gradually cooling the autoclave up to room temperature, the NiCoBDC (nickel, cobalt-terephthalate) MOF sample was collected (∼80 mg) as a powder and filtered with continuous washing of methanol. After washing the sample, it was dried in an air oven at 60 °C. Typically, 1.2 g of the already prepared NiCoBDC MOF was placed in a boat crucible and carbonized in a Vulcan furnace at 450 °C for 2 h (2 °C min^−1^) in air. The 0.350 g powder of NCC was collected and saved for catalytic applications.

### 2.3 Characterization

The infrared instrument of IRSpirit-T (Diamond ATR, Shimadzu) was used to confirm the functional group of ligands in MOF and the successful preparation of NCC in the range of 4000 to 400 cm^−1^. Thermal gravimetric analysis (TGA) was performed with an SDT thermal analyzer from room temperature to 1000 °C (TA instruments, USA) with a 20 °C min^−1^ ramp in N_2_ flow of 50 mL min^−1^. SEM-EDX of NCC was performed by using a ZEISS ULTRA PLUS-43-13 with connecting EDS by OXFORD X-MAX-50. Post-electro-catalyzed NCC analysis by field emission scanning electron microscopy (FESEM) aided with elemental analysis (JEOL JSM-7600F, 15 kV, Tokyo, Japan). Powder X-ray diffraction was performed by a Rigaku Miniflex diffractometer (Tokyo, Japan). X-ray photoelectron spectroscopy (XPS) was performed to analyze the oxidation state and elemental composition by the Scienta Omicron instrument (Taunusstein, Germany). Gas chromatography-mass spectrometry (GC-MS) analysis of samples was performed by using the GC-MS 7990B, 5977A (Agilent Technologies). Water splitting OER and HER was performed using the GAMMERY potentiostat (Reference-3000). BET analysis was performed by a Micromeritics ASAP 2020 surface area and porosity analyzer.

## 3. Results and discussion

**Fig. 1:**
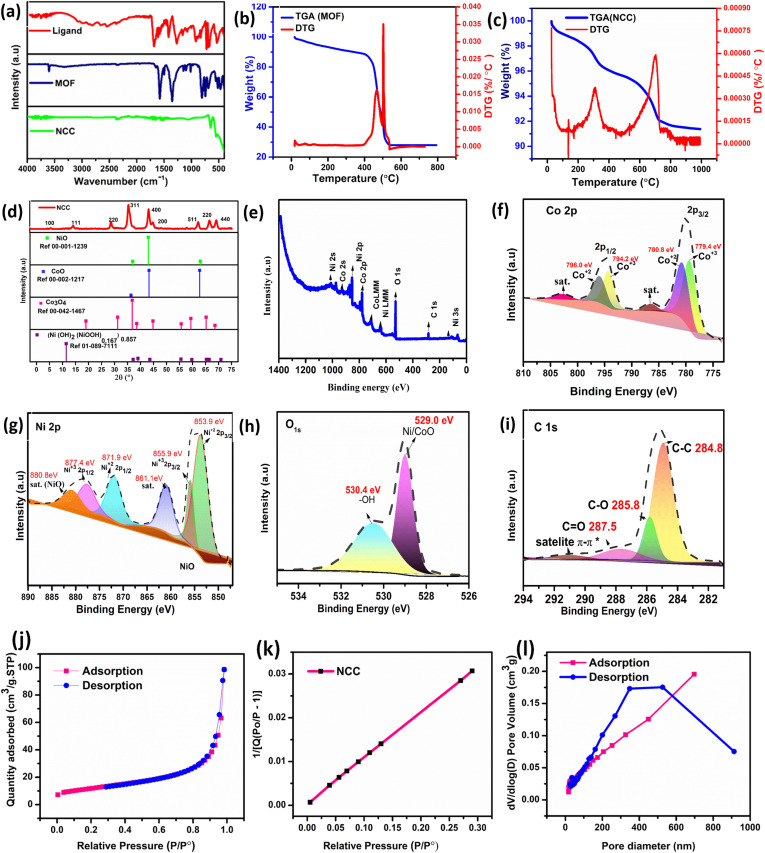
FTIR spectra of the ligand, Ni/Co-MOF, and NCC (a), thermal analysis of Ni/Co-MOF (b) and NCC (c), powder XRD of NCC with reference codes of NiO, CoO, and Co_3_O_4_ (d), XPS analysis (e–i), and BET surface analysis (j–l) of NCC.

**Fig. 2:**
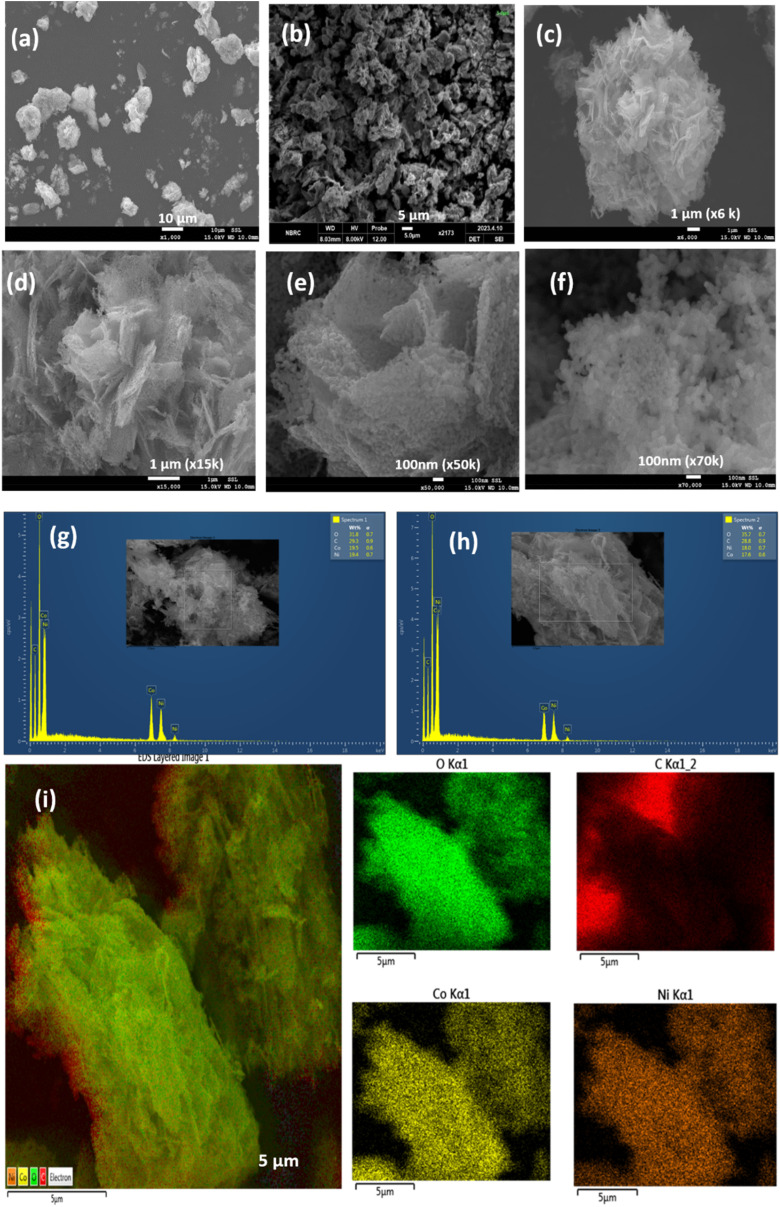
SEM images at different resolutions (a–f); EDX compositional distribution analysis (g and h); and elemental distribution mapping of Ni, Co, O, and C (i) for NCC.

The synthesis of materials was analyzed through different analytical techniques. Fourier transform infrared spectroscopic (FTIR) analysis, Fig. 1a, of bimetallic (Ni/Co) MOF, shows a carbonyl peak shifting to a lower frequency than that of the ligand's free carbonyl functional group. Strong peaks at 1577 and 1360 cm^−1^ correspond to the antisymmetric and symmetric stretching modes of the carbonyl functional moieties in the MOF, respectively. The absorption peak at 1497 cm^−1^ can be attributed to the C–H bending vibrations of the benzene rings. The peak at 744 cm^−1^ exhibited successful coordination between the metal and ligands. The nickel and cobalt oxide-doped carbon composite (NCC) depicts two corresponding peaks of stretching vibration for Ni–O and Co–O at 560 and 650 cm^−1^, respectively. TGA, Fig. 2b and c, of the MOF and NCC were performed to reveal two-step mass loss patterns. The weight loss of <200 °C is attributed to the loss of solvent. The observed stability of the MOF structure up to 400 °C is attributed to the interaction of two fully coordinated mixed metal nodes of nickel and cobalt and the absence of co-crystals or solvent coordination within the structure. There is a small weight loss (12%) at 405 °C. The major decomposition of MOF was carried out between 400–600 °C, with the DTG maximum at ∼450 °C, which is due to the loss of organic moieties from the MOF backbone structure, as shown in Table S1. However, a pyrolyzed sample of NCC has a small step decomposition as shown in Fig. 1c, with the mass loss leading to around 92% residual NCC as shown in Table S1. There is no major loss of mass, proving the stability of the sample up to 1000 °C. The crystal structure of NCC was analyzed by powder XRD (Fig. 1d), and it shows sharp peaks at 11.59°, 18.9°, 31°, 36°, 43°, 44°, 59°, 62°, and 65°. The observed peak at 11.59° closely matches with the (Ni(OH)_2_(NiOOH)_0.167_)_0.857_ peak at 11.43° with reference code (01-089-7111). The remaining 8 peaks best match with Co_3_O_4_ and NiO with references to ICDD-PDF codes (00-040-1191 and 00-001-1239, respectively). The data comparison with the reference codes revealed that the peaks at 43° and 62° belong to NiO, while the other peaks match well with Co_3_O_4_. The peaks' analysis of XRD with reference 00-002-1217 of CoO also best matched at the three peak positions of 36°, 43°, and 62°. The coexistence of both CoO and Co_3_O_4_ phases suggests partial oxidation of Co^2+^ to Co^3+^ during the synthesis process. The mixed-valence states may play a crucial role in enhancing the electrocatalytic activity due to the synergistic electronic and structural interactions.

The NCC exhibits a high degree of structural order within the crystal lattice as indicated by the crystallinity of 90.88% (Table S2). The crystallinity of NCC was measured by the area underneath the peaks in the XRD patterns. Meanwhile, the average crystallite size was measured to be 11 nm using the Scherrer equation, as shown in eqn (1) (Table S3), where *D* is the size of the crystals, *k* is the Scherrer equation shape constant (*k* = 0.9), *λ* is the X-ray wavelength, *θ* is the peak position, and *β* is the FWHM.^23^

1 *D* = *kλ*/*β* cos *θ*

The X-ray photoelectron spectroscopy (XPS) analysis revealed the atomic composition and valence states of the NCC composite. The XPS of the NCC composite (Fig. 1e) revealed the presence of Ni, Co, O, and C elements. The cobalt (2p) spectrum (Fig. 1f) shows two main peaks of Co 2p_3/2_ and Co 2p_1/2_ at around 780 and 795 eV, respectively, followed by satellite features in each case. These two peaks are further deconvoluted to Co^3+^ and Co^2+^ oxidation states, which indicate the existence of mixed valence cobalt species. Co 2p_3/2_ exhibits two peaks at 779.4 eV and 780.8 eV, attributed to Co^3+^ and Co^2+^, respectively. The Co 2p_1/2_ peaks of 794.2 and 796.0 eV correspond to Co^3+^ and Co^2+^, respectively. The peaks of Ni 2p are further split into characteristic peaks at 853.9 & 855.9, and 871.9 & 877.4 eV as depicted in Fig. 1g for 2p_3/2_ and 2p_1/2_, respectively, with their corresponding satellites. The corresponding O 1s peak at 529.0 in Fig. 1h was deconvoluted into two peaks of metal/oxide (NiO/Co_3_O_4_) and hydroxide O–H. The C 1s peak was measured at 284.8 eV and deconvoluted into three peaks of C–C, C–O, C=O, and a π–π* satellite as shown in Fig. 1i. The π–π* is due to the delocalized electron in aromatic rings, which can usually be detected a few eV away from the main corresponding energy. The nitrogen (N_2_) adsorption–desorption isotherms of the NCC sample have a type-IV profile with an H3-type of hysteresis loop, which is typical of mesoporous structures made up of slit-like pores caused by particle aggregation, as shown in Fig. 1j. The observed BET surface area is 41.60 m^2^ g^−1^. The BJH adsorption study revealed a surface area of 36.58 m^2^ g^−1^. Fig. 1k shows the pore size distribution between pore diameters and pore volumes of BJH adsorption and desorption. The average pore diameter for adsorption and desorption was measured at 16.31 and 18.61 nm, respectively. The NCC nanocomposite has a moderate surface area and consistent mesoporosity, which are ideal for diffusion-limited applications like catalysis.

To analyze the surface morphology of the NCC, SEM was performed (Fig. 2a–f). The surface morphology revealed clustering of the regularly shaped flakes, finally clustered/constructed as flowers (Fig. 2a–c). The SEM images (Fig. 2c and d) indicate that NCC has regular patterns of two-dimensional (2D) layers of ∼100 nm thickness, which are clustered with each other. These 2D nanolayers are arranged in parallel and embedded into each other at different angles within a bunch of macro clusters. The layers are built from nano-spherical particles of mesoscopic diameters in the range of ∼30 nm (Fig. 2e and f). This morphology is beneficial to increase the surface area of the material, enhance the penetration of small molecules, and thus promote the physicochemical features of the NCC material. NCC contains cross-linked nanoparticle layers that are interconnected to each other in different directions, with space between adjoining nanospheres. This two-dimensional nanoarray structure not only enables efficient exposure of active sites, but also facilitates mass transfer. These mesospheres, building nanolayers self-assembled as macro-sized clusters/aggregates, are further analyzed by EDS (Fig. 2g and h). These SEM-EDS graphs reveal the contents of O, C, Co, and Ni in the samples with their respective weights (%) ranges: 31.8–35.7, 28.8–29.3, 17.6–19.5, and 18–19.4 wt%. The compositional distribution of the NCC was further investigated by the EDS elemental mapping shown in Fig. 2i, revealing that Co, Ni, O, and C are uniformly distributed throughout the entire morphology.

## 4. Catalytic activities

### 4.1 Carbon dioxide cycloaddition

Cycloaddition of CO_2_ to epoxides represents one of the very promising industrial processes operating under ambient temperatures and pressure conditions. The value-added cyclic carbonates are important chemical products that are used for a variety of applications. Enhanced activity and selectivity for the coupling reaction of CO_2_ and epoxides can be achieved through the use of effective catalysts. Here, CO_2_ is reacted with epoxides in the presence of NCC at a certain temperature (50–100 °C), and the epichlorohydrin substrate was loaded in the reactor. The reaction was also performed without a co-catalyst at 100 °C, as shown in entry 1 in Table 1, and it gave an appropriate conversion of 74.60% with 38.35% selectivity. It is evident that using TBAB as a co-catalyst enhances the cycloaddition activity of epoxide; however, conversion without the use of co-catalysts was considerable. At the lower temperature down to 50 °C (entry 2 of Table 1), the reaction yield was 94.5% with 76.71% selectivity. In the reaction carried out at 100 °C in the presence of catalyst and TBAB, excellent conversion and selectivity to the cyclic carbonate product were achieved at 99.96% and 98.5%, respectively, under the same conditions, as shown in entry 3 in Table 1. The recovered catalyst was reused affording 99.1% activity (entry 4 in Table 1) under same reaction conditions. The cycloaddition of CO_2_ proceeds efficiently under the optimized conditions of 100 °C for 24 h, as shown in Scheme S1, ensuring that the catalytic activity of NCC for CO_2_ is more significant than that of different previously reported Ni–Co-based catalysts, as shown in Table 1 (entries 5–8). The FTIR analysis of the recovered MOF was performed to confirm the 100% recovery of the catalyst with no change. The recovery of the catalyst was further confirmed by FTIR, as shown in Fig. S1, and XRD in Fig. S2. Gas chromatographic analysis was performed to analyze the reaction mixture.

**Table 1:** Different Ni- and Co-based catalyst activities for the cycloaddition of CO_2_ through different substrates

Catalyst	Substrate	Co-catalyst	Conversion (%)	Selectivity (%)	Conditions (*T*/°C, *P*_CO_2__/MPa, Cat./mg, & *t*/h)				Ref.
NCC	ECH^a^	—	74.6	38.4	100	0.2	10	24	This work
NCC	ECH	TBAB^d^	94.6	76.7	50	0.2	10	24	
NCC	ECH	TBAB	99.9	98.5	100	0.2	10	24	
NCC^f^	ECH	TBAB	99.1	99.1	100	0.2	10	24	
NiCoBDC	ECH	TBAB	37	—	80	1.2	—	12	24
Ni-BDCNs	SO^b^	TBHP^e^	87	—	80	1	40	12	25
Ni-BTC	EP^c^	TBAB	45	—	90	0.1	60	—	26
Co@CN	ECH	—	81.7	—	150	0.3	16	1.5	27

a Epichlorohydrin.

b Styrene oxide.

c Ethoxypropanol.

d Tetrabutylammonium bromide, and.

e *Tert*-butyl hydroperoxide.

f Recycled.

The probable mechanism is that the metal centers in NCC act as Lewis acid sites to coordinate with the O atom of epoxide, which can activate the epoxy rings. The nucleophilic Br^−^ from *n*Bu_4_NBr attacks the less hindered C atom of the epoxy rings to open the ring. Then, CO_2_ molecules enter the opened epoxy ring to interact with the oxygen anion and obtain an alkyl carbonate salt (Scheme S2).

### 4.2 Electrocatalysis

#### 4.2.1 Electrochemical measurements

Electrochemical measurements were performed in a standard three-electrode system with Ag/AgCl as the reference electrode, Pt wire serving as the counter electrode, and nickel foam (NiF) as the working electrode. The electrocatalyst inks were made by dispersing 5 mg of the catalysts in a 1 mL solution mixture of ethanol and isopropyl alcohol (2 : 8) and 5 μL of 5% Nafion solution. The suspension was then drop-cast onto the NiF electrode (1 × 1 cm^2^) with a catalyst mass of ∼1 mg cm^−2^. The catalyst-loaded NiF was dried at 60 °C and used for electrocatalysis in the given electrolytic media. The formula *E*_RHE_ = *E*_Ag/AgCl_ + 0.197 + 0.0597 × pH was used to calibrate all of the potentials in this study as *V vs.* RHE. Different techniques like LSV, CV, EIS, and bulk electrolysis were performed.

#### 4.2.2 Oxygen evolution reaction (OER)

**Fig. 3:**
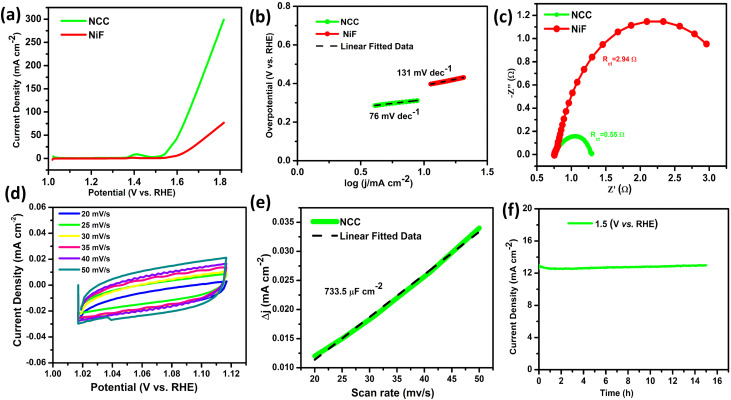
Oxygen evolution reaction (OER) in 1 M KOH. (a) LSV analysis at 10 mV s^−1^, (b) Tafel plot analysis, and (c) EIS spectra of NCC and NiF measured at 1.317 V *vs.* RHE as a Nyquist plot at 0.1–100 000 Hz. (d) Non-faradic run of NCC under different scan rates (20–50 mV s^−1^). (e) The double-layer capacitance (*C*_dl_) graph, derived from the non-faradaic region, for the NCC (f) stability run of NCC around 15 h.

The electrochemical performance of the catalyst was carried out in 1 M KOH using a conventional three-electrode system at standard room temperature. OER analysis was performed through the polarization curve of linear sweep voltammetry (LSV) (Fig. 3a) for NCC, the low overpotentials *vs.* RHE (*η*_10_ = 310 and *η*_50_ = 370 mV) as compared with bare NiF (*η*_10_ = 390 and *η*_50_ = 510 mV) at 10 and 50 mA cm^−2^, respectively. The corresponding Tafel slope value for NCC is 76 mV dec^−1^, which is much lower than that of the bare NiF (131 mV dec^−1^), in Fig. 3b. The Tafel slope value supports the better reaction kinetics and efficient charge transfer, where both initial electron transfer and subsequent intermediate steps contribute to the rate-determining step. Electrochemical impedance spectroscopy (EIS) was performed for the Nyquist plots' semicircles, and the observed data were fitted with an equivalent circuit (Fig. S3), which is composed of *R*_s_ (solution resistance), a constant-phase element, and *R*_ct_ (charge-transfer resistance). Values of *R*_s_ and *R*_ct_ are dependent on the electrocatalytic activity of the material, and a lower value corresponds to faster reaction kinetics. The EIS result depicted in Fig. 3c shows that *R*_ct_ has a value of 0.55 and 2.94 Ω for NCC and NiF at 1.317 V *vs.* RHE under 0.1–100 000 Hz, respectively. The small charge resistance value indicates that this catalyst is the best candidate for OER due to the high conductivity of charge and smooth flow of electrons. Cyclic voltammetry (CV) was recorded at different scan rates (Fig. S4a) in both Faradaic and non-Faradic regions. The CV was also run in non-Faradic regions for the double-layer capacitance (*C*_dl_) shown in Fig. 3d for NCC. The *C*_dl_ parameter was calculated from the relationship between the scan rate and current density difference between the anodic and cathodic regions (*j*_2_ − *j*_1_) evaluated from the CV data. Hence, the linear fit was applied to actual data, and the slope value was recorded at 733.5 μF cm^−2^ for NCC as shown in Fig. 3e, which was significantly higher under the given conditions. The electrochemical surface areas (ECSAs) calculated for NCC (18.25 cm^2^) indicate the higher surface area of the NCC catalyst. CPE analysis reveals stability at a constant potential of 1.58 V *vs.* RHE, with a consistently high current density exhibited continuously for 15 hours (Fig. 3f), demonstrating excellent durability in alkaline solution. All these electrochemical studies of NCC reveal its better OER activity compared to that of the bare NiF and other Ni, Co-based catalysts in Table 2.

**Table 2:** Different Ni and Co-based catalyst activities for the oxygen evolution reaction (OER) in 1 M KOH

Catalyst	Support/electrode	Overpotential (*η*_10_/mV)	Tafel slope (mV dec^−1^)	Stability (h)	Ref.
NCC	NiF^a^	310	76	15	T.W.^e^
C@NiCo-12	GC^b^	330	157	10	29
NiCo-NCS	NiF	310	98	10	30
0.5 AgSA-NiCo	CC^c^	192	39	500	31
NiCo/NiCoP	NiF	117	44.8	50	32
Ni/Co/Co_3_O_4_@C	GC	246	—	24	33
Ni-BDC@NF	NiF	436	—	45	34
NF@Ni/C	NiF	265	57	50	35
Mn-Co_1.29_Ni_1.71_O_4_	NiF	334.3	76.7	14	36
Co-MOF (X_2_)	NiF	180	66.4	20	37
NiCoO_2_/NiCo@C	CP^d^	329	61.88	15	38

a Nickel foam.

b Glassy carbon.

c Carbon cloth.

d Carbon paper.

e This work.

#### 4.2.3 Hydrogen evolution reaction (HER) in alkaline media

**Fig. 4:**
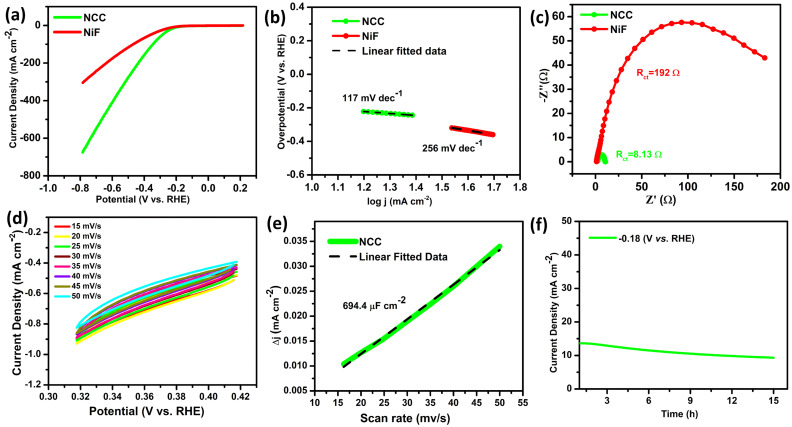
Hydrogen evolution reaction (HER) in 1 M KOH. (a) LSV polarization curves at 10 mV s^−1^, (b) corresponding Tafel plot of NiF and NCC at 10 mV s^−1^, (c) Nyquist plot with a semi-circular shape measured at 0.0112 V *vs.* RHE at 0.1–100 000 Hz for both NCC and NiF, (d) non-Faradic CV at different scan rates of (15–50 mV s^−1^), (e) *C*_dl_ graph of NCC, and (f) stability analysis of NCC for 15 hours.

The NCC catalyst was run under alkaline conditions (1 M KOH) for HER activity. The polarization curves (Fig. 4a) show the low overpotential for NCC (*η*_10_ = 200 & *η*_50_ = 230 mV) and bare NiF (*η*_10_ = 288 & *η*_50_ = 362). The Tafel slope (Fig. 4b) for NCC was evaluated (117 mV dec^−1^), and found to be much lower than that of bare NiF (256 mV dec^−1^) and other nickel and cobalt-based catalysts (as shown in Table 3). It follows that shown in the Volmer reaction at ≈120 mV dec^−1^; hence, the H–OH bond breaks to form the H atom for adsorption.^28^ The EIS analysis of NiF and NCC (Nyquist plots) clearly shows a large difference in charge transfer resistance. The NiF shows a very high *R*_ct_ value of 192 Ω at 0.0112 V *vs.* RHE with 0.1 to 100 000 Hz, which gives poor charge transfer and poor conductance. However, the catalyst sample NCC exhibits a very low *R*_ct_ (8.13 Ω, Fig. 4c), which facilitates the reaction kinetics. The performance of a catalyst is based on the active sites and the surface area, so it is very important to evaluate the ECSA to explore the available active sites. Faradaic and non-Faradic cyclic voltammetry was run under different scan rates for HER in 1 M KOH, as shown in Fig. S4b. However, the non-Faradic CVs (Fig. 4d) were used to find the *C*_dl_ of NCC, which was obtained as 694.4 μF cm^−2^, and their corresponding ECSA values (*C*_dl_/*C*_s_) were measured at 17.35 cm^2^. Evaluating the catalytic performance of NCC for HER activity durability and stability is a crucial parameter. Hence, the stability test was performed, demonstrating that the NCC sample shows robustness and stability at a constant potential of 0.18 V *vs.* RHE with almost no change in current density at ∼12 mA cm^−2^ for 15 h in Fig. 4f. The HER activity of NCC as a bimetallic catalyst is very good compared with that of other electrochemical catalysts, as shown in Table 3. MoN/Co_2_N hybrid nanosheets integrated on Cu foam also show how hierarchical structures and N-doped carbon layers carried out seawater splitting with a low overpotential of 1.7 V@*η*_100_ in 1 M KOH with significant durability, much like our pyrolyzed Ni–Co-BDC-derived catalyst NCC. This further demonstrates how multi-dimensional architectures and heteroatom-doped carbon frameworks help stabilize active sites under challenging electrolysis conditions.^39^

**Table 3:** Different Ni and Co-based catalyst activities for the HER in different (alkaline and acidic) electrolytes

Catalysts	Support	Electrolyte	Overpotential (*η*_10_/mV)	Tafel slope (mV dec^−1^)	Stability (h)	Ref.
NCC	NiF	1 M KOH	200	117	15	T.W.^d^
NCC	NiF	0.1 M H_2_SO_4_	118	47	10	
NiCoO_2_@NC	—	1 M KOH	94	125	50	41
FeOOH@NiCO_2_O_4_	NiF	1 M KOH	146	41.3	10	42
NiCO_2_O_4_ cuboidal	HMC^a^	1 M NaOH	110	49.7	32	43
NiCo/NiCO_2_S_4_@NiC	NiF	1 M KOH	176	58.2	28	44
NiCo alloy-300	NiF	1 M KOH	156	82.7	12	45
NiCoP	CC	0.5 M H_2_SO_4_	48	38.5	24	46
Mn-Co_1.29_Ni_1.71_O_4_	NiF	1 M KOH	203	113.6	14	36
Co MOF (X_2_)	NiF	1 M KOH	151.7	44.4	20	37
NiCo_2_S_4_	GCE	0.5 M H_2_SO_4_	120	56	30	47
Ni-Co-MoS_2_	CP^b^	0.5 M H_2_SO_4_	100	45	24	48
NiS_2_/NS	GS^c^	0.5 M H_2_SO_4_	105	40	12	49
Ni(OH)_2_/CoNi_2_S_4_/NF	NiF	0.5 M H_2_SO_4_	90	38	24	50
Ni/NiS-3@CP	CP	0.5 M H_2_SO_4_	125	180	20	51
Cu/Ni/MFI-PZ/GC	GCE	0.5 M H_2_SO_4_	385	96	2	52
Ni-6W	SP^e^	1 M KOH	668@*η*_50_	168	72	53

a Hollow microcuboids.

b Carbon paper.

c Graphite substrate.

d This work, and.

e Steel plate.

The OER and HER performance of pristine MOF NiCoBDC in alkaline media are shown for comparison, which includes the LSV and corresponding Tafel slopes (Fig. S5). As evident from these graphs, the composite NCC catalytic activity is significantly better than that of the bare NiF and parent MOF.

#### 4.2.4 HER in acidic media

**Fig. 5:**
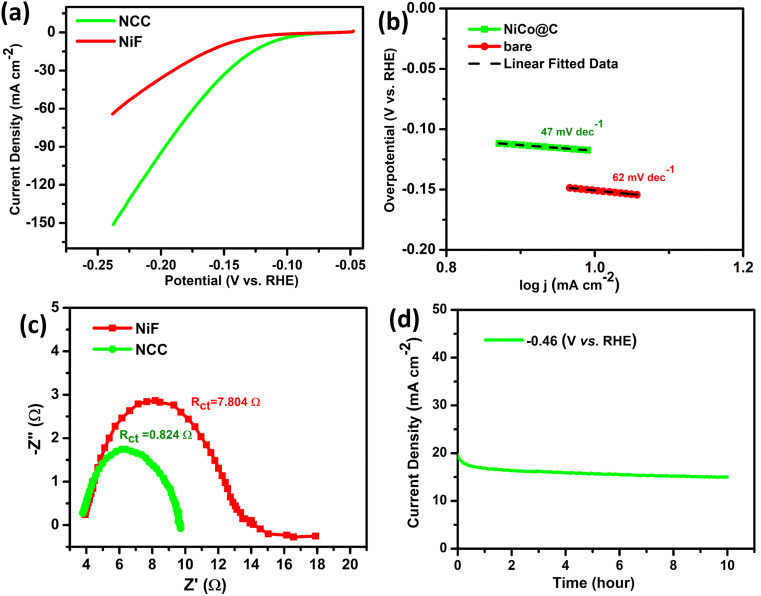
HER in 0.1 M H_2_SO_4_. (a) LSV at 10 mV s^−1^, (b) Tafel plots, (c) Nyquist plots at −0.3 V under 0.1–100 kHz, (d) and stability analysis of the sample at the fixed potential (−0.46 V *vs.* RHE) for 10 h.

The electrochemical activity of HER for NCC was also characterized in a mild acidic solution of 0.1 M sulphuric acid (H_2_SO_4_) with a low overpotential (*η*_10_ = 118 & *η*_50_ = 166 mV) as compared to bare NiF (*η*_10_ = 150 & *η*_50_ = 220 mV) at the expense of −10 and −50 mA cm^−2^, respectively (Fig. 5a). The Tafel slope of NCC was (47 mV dec^−1^) much lower than that of NiF (62 mV dec^−1^) as shown in Fig. 5b, which indicates that HER proceeds *via* the Volmer–Heyrovsky mechanism, with electrochemical desorption of the Heyrovsky step as the rate-determining step. The charge transfer at the electrode–electrolyte interface occurs by reducing the energy barrier. The EIS plots of NCC and bare NiF in Fig. 5c revealed significant efficiency with low charge transfer resistance (0.824 and 7.804 Ω, respectively). The Faradaic and non-Faradic CV scans were run for NCC in 0.1 M H_2_SO_4_, as shown in Fig. S4c. The stability test was conducted by the fixed potential electrolysis of −0.462 V *vs.* RHE for 10 hours to evaluate the long-term stability, as depicted in Fig. 5d. The electrode exhibits stable current density at 16 mA cm^−2^ with very minor fluctuations. The 0.1 M H_2_SO_4_ is significantly milder and even less aggressive toward nickel. The NCC experiences no physical or chemical deterioration of the NiF substrate, and the electrochemical performance remained stable throughout the measurements. These findings collectively support the practical stability of NiF in the employed acidic electrolyte. This stability aligns well with the acid tolerance of nickel-based systems, as exemplified by Ni foam electrodes, which have been reported to sustain high HER activity for over 5 days in acidic media under comparable conditions.^40^ Hence, all these results demonstrate that NCC is the best candidate for HER in both acidic and basic conditions.

#### 4.2.5 Post electrolysis stability

**Fig. 6:**
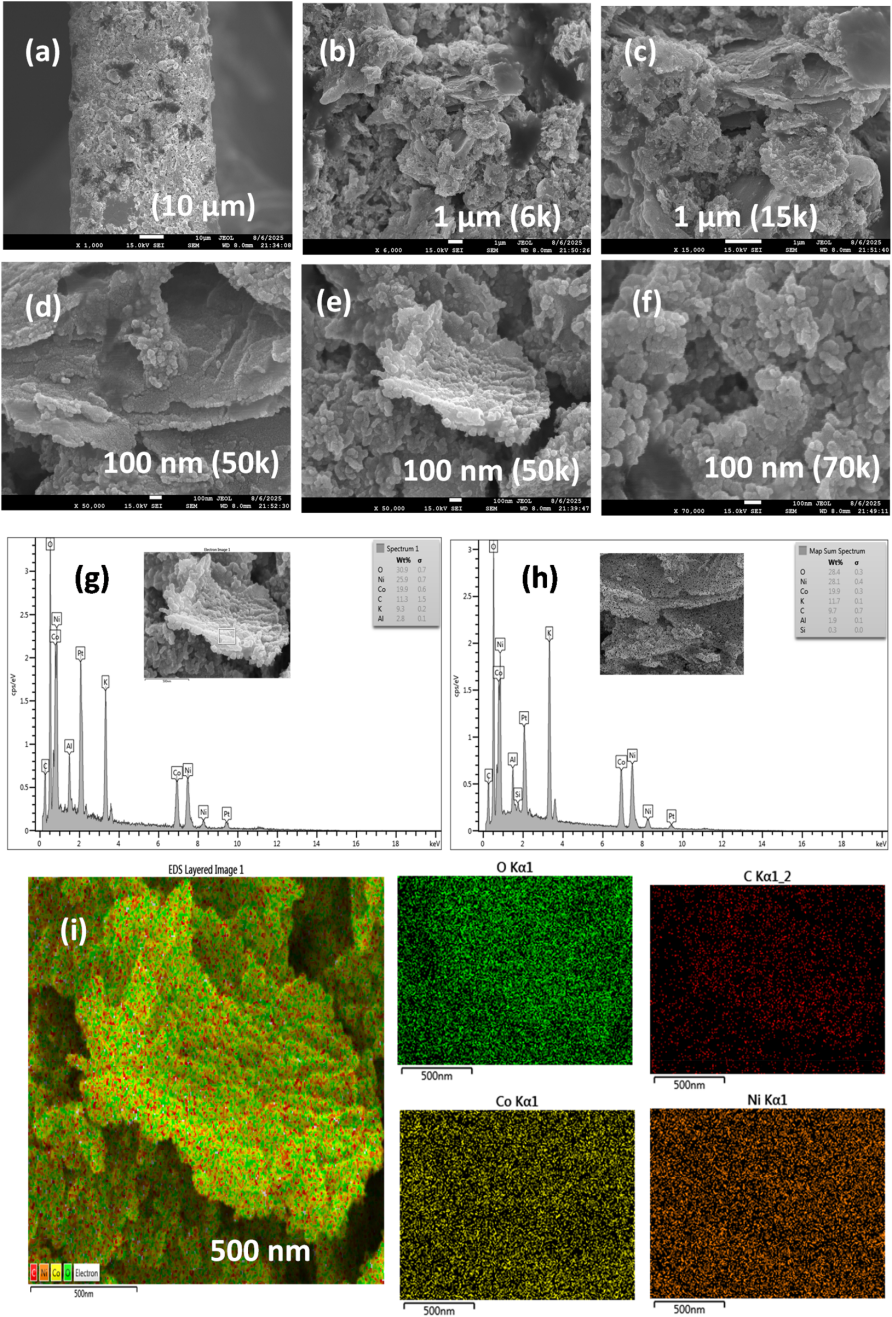
Post-electrocatalyzed NCC analysis after 1000 CVs at 100 mV s^−1^ in 1 M KOH for the OER/HER: (a–f) FESEM; (g and h) EDS elemental composition; and (i) elemental distribution mapping as coloured spots with respect to Ni, Co, O, and C.

Morphological and compositional (EDS, and elemental mapping) analysis of NCC after extensive 1000 CV cycles (500 CVs for OER + 500 CVs for HER) at 100 mV s^−1^ in 1 M KOH was performed *via* fast emission scanning electron microscopy (FESEM). When compared to the pristine NCC catalyst in Fig. 2a–f, the surface morphology of the NCC underwent negligible changes, *i.e.*, declustering from the flower shaping of the 2D sheets/flakes of the nanoparticle, as observed by the FESEM images (Fig. 6a–f) at the same magnifications. This is probably due to the ink preparation, loading, and electrocatalyzing process. However, the nanoparticle morphology remains the same in the decorated form of 2D flakes/sheets. Notably, the particle size (as observed from ×50 000–70 000 magnifications, Fig. 6e and f) is ∼30 nm, which is consistent with the pre-electrolysis NCC's nanoparticles, indicating excellent morphological stability during electrochemical activity. Even after prolonged HER operation, the EDS spectra (Fig. 6g and h) demonstrate the preservation of important constituent elements, including the uniform distribution of Ni, Co, C, and O. The surface contains some species as indicated by the presence of potassium (K) from the electrolyte and a discernible rise in oxygen contents, which is probably caused by contact with hydroxide ions during cycling. The material's chemical stability under electrochemical circumstances is highlighted by the elemental atomic ratio remaining consistent with the anticipated stoichiometry. The corresponding peaks of some other species most likely come from the sample treatment for scanning analysis. Elemental color mapping was done in order to evaluate the distribution of elements even more (Fig. 6i). A consistent distribution of Ni (green), Co (yellow), C (red), and O (orange) over the sample surface is seen from the maps. This homogeneity supports the NCC structural stability and compositional durability by indicating that there is no discernible leaching or agglomeration of active ingredients during prolonged storage.

## 5. Conclusions

The hybrid nanomaterial was synthesized from the MOF precursor NiCoBDC through pyrolysis at 450 °C, resulting in a highly active nanocomposite for catalysis. Structural and morphological analyses (FTIR, TGA, PXRD, SEM, EDX, and XPS) confirmed the successful synthesis of the nanocomposite (NCC) as a metal oxide-doped, carbon-derived structure. The NCC sample exhibits significant CO_2_ fixation activity (99%) at a mild temperature of 50 °C and 1 atm pressure. It demonstrates low overpotentials (118–310 mV) and low Tafel slopes (47–117 mV dec^−1^) under both alkaline (1 M KOH for OER/HER) and acidic (0.1 M H_2_SO_4_ for HER) conditions. The nanocomposite is conductive, as shown by the Nyquist plots, due to embedded nickel and cobalt species. The ECSA is very high, indicating excellent electrocatalytic activity for OER and HER. The NCC sample demonstrates robustness and stability over long-term use for up to 10–15 hours with minimal fluctuation in current density. As a result, NCC functions as a bifunctional catalyst for both CO_2_ fixation and water splitting. Overall, this study presents a promising strategy for developing MOF-derived carbon hybrid nanomaterials as electrocatalysts to tackle challenges in sustainable energy and CO_2_ management.

## Conflicts of interest

The authors declare no conflicts of interest.

## Data Availability

Additional information/data can be provided upon reasonable request. The data supporting the findings of this study are available within the article and its SI. Supplementary information: Characterization results such as TGA, PXRD, FTIR, and electrochemical mesurements. See DOI: https://doi.org/10.1039/d5ra04999g.
