# Correction: Dual-functional Ni and Co oxide-doped carbon nanocomposite: an effective catalyst for electrochemical water splitting and CO_2_ utilization

**DOI:** 10.1039/d5ra90110c

**Published:** 2025-09-26

**Authors:** Misbah Zia, Zahoor Ahmad, Khurram S. Munawar, Zafar A. K. Khattak, Hamid Raza, Hira Idrees, Hussein A. Younus, Muhammad Ashraf Shaheen, Nazir Ahmad

**Affiliations:** a Institute of Chemistry, University of Sargodha Sargodha-40100 Pakistan; b Department of Chemistry, Government College University Lahore Lahore-54000 Pakistan dr.nazirahmad@gcu.edu.pk; c Department of Chemistry, Faculty of Science, Fayoum University Fayoum 63514 Egypt hay00@fayoum.edu.eg; d Department of Chemistry, Faculty of Science, University of Engineering and Technology Lahore Pakistan; e Department of Chemistry, University of Mianwali Mianwali 42200 Pakistan; f Department of Chemistry, University of Buner Swari, Buner 19281 Pakistan; g Department of Chemistry, University of Management and Technology Lahore 54770 Pakistan; h Nanotechnology Research Center, Sultan Qaboos University P.O. Box 17, 123 Al-Khoud Oman; i Faculty of Allied Health Sciences, Superior University Lahore Pakistan

## Abstract

Correction for ‘Dual-functional Ni and Co oxide-doped carbon nanocomposite: an effective catalyst for electrochemical water splitting and CO_2_ utilization’ by Misbah Zia *et al.*, *RSC Adv.*, 2025, **15**, 32667–32678, https://doi.org/10.1039/D5RA04999G.

The authors regret that the name of one of the authors (Hussein A. Younus) was shown incorrectly in the original article. In addition, one of the affiliations (affiliation *i*) was also incorrectly shown. The corrected author list and affiliations are as shown here.

The Royal Society of Chemistry apologises for these errors and any consequent inconvenience to authors and readers.

