# Correction: Dual-functional Ni and Co oxide-doped carbon nanocomposite: an effective catalyst for electrochemical water splitting and CO_2_ utilization

**DOI:** 10.1039/d6ra90064j

**Published:** 2026-07-06

**Authors:** Misbah Zia, Zahoor Ahmad, Khurram S. Munawar, Zafar A. K. Khattak, Hamid Raza, Hira Idrees, Hussein A. Younus, Muhammad Ashraf Shaheen, Nazir Ahmad

**Affiliations:** a Institute of Chemistry, University of Sargodha Sargodha-40100 Pakistan; b Department of Chemistry, Government College University Lahore Lahore-54000 Pakistan dr.nazirahmad@gcu.edu.pk; c Department of Chemistry, Faculty of Science, Fayoum University Fayoum, 63514 Egypt hay00@fayoum.edu.eg; d Department of Chemistry, Faculty of Science, University of Engineering and Technology Lahore Pakistan; e Department of Chemistry, University of Mianwali Mianwali 42200 Pakistan; f Department of Chemistry, University of Buner Swari Buner 19281 Pakistan; g Department of Chemistry, University of Management and Technology Lahore 54770 Pakistan; h Nanotechnology Research Center, Sultan Qaboos University P. O. Box 17, 123 Al-Khoud Oman; i Faculty of Allied Health Sciences, Superior University Lahore Pakistan

## Abstract

Correction for ‘Dual-functional Ni and Co oxide-doped carbon nanocomposite: an effective catalyst for electrochemical water splitting and CO_2_ utilization’ by Misbah Zia *et al.*, *RSC Adv.*, 2025, **15**, 32667–32678, https://doi.org/10.1039/D5RA04999G.

The authors regret that the raw experimental XPS spectra were inadvertently omitted from the published XPS deconvolution figures (Fig. 1(f–i)). The corrected Fig. 1 is shown below. The authors confirm that no experimental data, peak assignments, fitting parameters, interpretations, or conclusions of the manuscript are affected by this revision because no changes have been made to the peak assignments or interpretation of the XPS results.

**Fig. 1:**
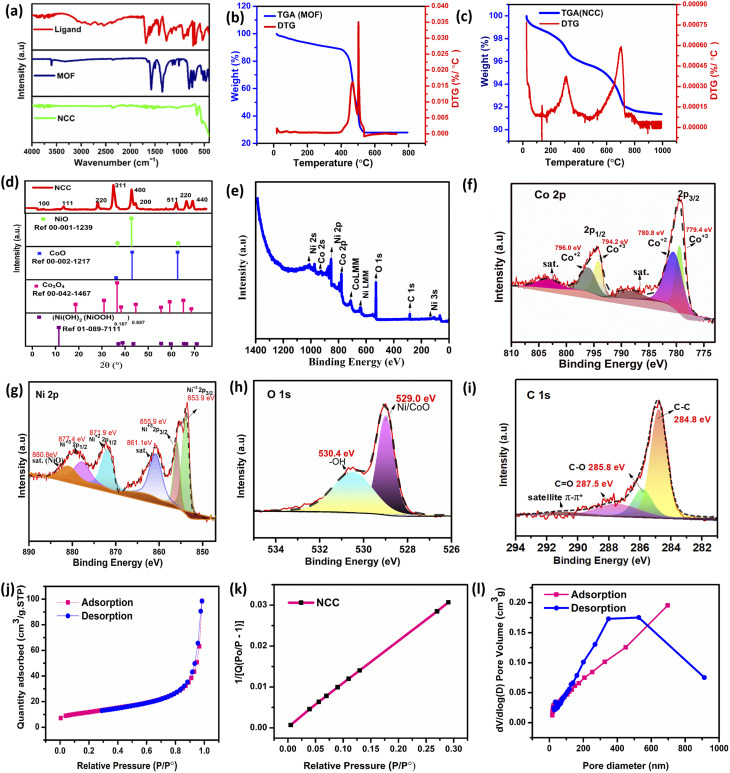
FTIR spectra of the ligand, Ni/Co-MOF, and NCC (a), thermal analysis of Ni/Co-MOF (b) and NCC (c), powder XRD of NCC with reference codes of NiO, CoO, and Co_3_O_4_ (d), XPS analysis (e–i), and BET surface analysis (j–l) of NCC.

The updated Fig. 1 has been reviewed by an independent expert, and this correction does not alter the conclusions presented in this article.

The Royal Society of Chemistry apologises for these errors and any consequent inconvenience to authors and readers.

